# Bromelain in Burn Care: Advancements in Enzymatic Debridement and Patient Outcomes

**DOI:** 10.3390/ebj5040039

**Published:** 2024-12-12

**Authors:** Eliza-Maria Bordeanu-Diaconescu, Sabina Grama, Andreea Grosu-Bularda, Adrian Frunza, Mihaela-Cristina Andrei, Tiberiu-Paul Neagu, Ioan Lascar

**Affiliations:** 1 Department 11, Discipline Plastic and Reconstructive Surgery, University of Medicine and Pharmacy Carol Davila, 050474 Bucharest, Romania; eliza.diaconescu@umfcd.ro (E.-M.B.-D.); andreea.grosu-bularda@umfcd.ro (A.G.-B.);; 2 Burn Centre, Emergency Clinical Hospital of Bucharest, 014461 Bucharest, Romania

**Keywords:** bromelain, NexoBrid^®^, enzymatic debridement, burns, hand burns, facial burns, burn wounds

## Abstract

The management of severe burns is a complex process that requires a multidimensional approach to ensure optimal healing of burn wounds, minimize complications, and improve the prognosis of patients. Surgical debridement is considered the gold standard for removing necrotic tissue; however, this approach involves risks such as bleeding, the potential removal of viable tissue during excision, and technical challenges in complex anatomical areas. Recent advancements highlight the role of enzymatic debridement using NexoBrid^®^, which offers a less invasive alternative to surgical excision while having the ability to selectively debride necrotic tissue and preserve viable tissue. NexoBrid^®^ has shown efficacy in reducing debridement time, minimizing the need for additional surgeries, and improving overall wound healing outcomes. This review discusses the clinical indications, advantages, and considerations for choosing between surgical and enzymatic debridement. Emerging studies suggest the potential for enzymatic debridement to be safe and effective even for larger burn areas, making it a promising option in modern burn care. However, ongoing evaluation and integration into clinical protocols will be essential to fully realize its benefits in specialized burn treatment and to establish protocols.

## 1. Introduction

Severe burns are characterized by expansive tissue damage and often necessitate complex therapeutic approaches. The management of extensive burn wounds involves initial stabilization of the patient, including fluid resuscitation to address shock and prevent hypovolemia, followed by careful wound care, which involves debridement to remove damaged tissue and prevent infection, and the application of advanced dressings or grafts to promote healing. Pain management, nutritional support, and rehabilitation are important components of the treatment process to support recovery and reduce long-term complications. In addition to these acute care measures, continuous monitoring for complications such as infections, organ dysfunction, and psychological impacts is essential. Burn care specialists, surgical specialists, and physical therapy specialists are part of a multidisciplinary team which is essential for improving the care of patients with extensive burns [1,2,3].

In the 1970s, pioneering burn surgeon Zora Janzekovic introduced the technique of early tangential excision. This technique involves the early removal of necrotic tissue from burn wounds through thin, precise layers of excision, helping to preserve as much viable tissue as possible. Janzekovic’s innovation significantly reduced the risks associated with infection and sepsis, the major causes of death in burn patients at the time. Additionally, early tangential excision helped improve wound healing outcomes and shorten the length of stay for burn patients. This approach became the gold standard in modern burn care and is still the standard of care nowadays [4,5,6].

Effective management of burn wounds is important for both functional and aesthetic recovery in burn patients. Early debridement can be accomplished through various surgical techniques, including tangential excision, as well as hydrosurgery and enzymatic debridement. After debridement, depending on burn depth, burn wounds heal by secondary reepithelization or are covered with autografts (the standard of care for deep partial-thickness or full-thickness burn wounds). In patients lacking donor areas for autografts, temporary coverage of burn wounds can be also obtained using allografts or xenografts. Permanent skin substitutes include a variety of options such as dermal templates that require later coverage with thin split-thickness skin grafts or epidermal layers, cultured epidermal autografts, autologous skin cell suspension, and fully bioengineered dermo-epidermal skin substitutes. Negative Pressure Wound Therapy (NPWT) is increasingly utilized in burn management due to its ability to remove exudate, reduce bacterial load, and enhance the adherence and integration of skin grafts [7,8,9,10].

Although surgical excision remains the standard practice for removing necrotic tissue and preparing the wound bed for grafting, there is increasing interest in using enzymatic debridement as a complementary therapy for burn wound management [11,12,13]. Surgical excision for burn wounds has several drawbacks, including significant blood loss during surgery, being an invasive procedure that often requires general anesthesia, potential scarring and cosmetic issues, and the need for a surgeon experienced in burn care who can distinguish between viable and non-viable tissues. Regardless of the instruments used (Goulian or Watson knives or dermatomes), tangential excision leads not only to the debridement of necrotic tissues but also the debridement of healthy skin and tissues with prolonged recovery times. Even experienced surgeons face the risk of over-excision, particularly in anatomically complex areas, when performing surgical debridement for burn wounds [14,15,16]. On the other side, surgical excision followed by coverage with autografts is a more straightforward approach, with well-defined stages of wound healing, graft acceptance, and rehabilitation.

Over time, a range of products have been investigated for their effectiveness in wound debridement, including Papain-Urea, Sutilains (derived from Bacillus subtilis cultures), trypsin, vibriolysin, enzymes from papaya and fig trees, blow fly larvae, pyruvic acid, and phosphoric acid. However, these products have demonstrated either very low efficacy or significant side effects, leading to their limited use in clinical practice [17,18,19,20].

Bromelain has its roots in ancient South American civilizations, where indigenous communities used the pineapple plant (Ananas comosus) for treating ailments like digestive disorders, inflammation, and wounds. Its modern history began in the late 19th century when researchers started investigating the enzyme’s medicinal potential, eventually recognizing its therapeutic properties for various medical applications [21]. Bromelain is a major sulfhydryl proteolytic enzyme found in pineapple plants that belongs to the cysteine protease group. Two different enzymes are extracted from pineapples, fruit bromelain and stem bromelain, that differ in their structure, biological activity, and physicochemical properties. Among them, only stem bromelain is used in medicine [21,22]. Bromelain possesses anti-inflammatory and analgesic properties and has demonstrated the ability to selectively digest necrotic tissue while preserving healthy tissue, leading to faster and less painful wound healing [23]. Bromelain-based enzymatic debridement has emerged as a reliable alternative to surgical eschar removal, showing particular effectiveness in treating mid- to deep dermal and mixed-depth burns, with a growing number of burn centers reporting successful outcomes [24].

In 2012, NexoBrid^®^ (MediWound Ltd., Yavne, Israel), a Bromelain-based debridement concentrate of proteolytic enzymes (anacaulase-bcdb, thiol-endopeptidases, peroxidases, phosphatases, glucosidases, and cellulases) derived from pineapple stems, with a standardized composition (0.09 g of bromelain per gram of product), was introduced to the European market following promising outcomes in several studies that highlighted its improved selectivity in preserving vital tissue and its efficiency in reducing the time required for complete debridement [25,26]. Following its approval from the European Medicines Agency (EMA) in 2012, NexoBrid^®^ was granted U.S. Food and Drug Administration (FDA) approval in 2022.

NexoBrid^®^ offers a minimally invasive alternative to surgical debridement for removing thermally damaged tissue. Studies have shown that NexoBrid^®^ significantly shortens the time needed for complete debridement, reduces the need for additional surgery, and minimizes the total area excised and grafted. It effectively minimizes bleeding and allows for treatment to be performed immediately after the injury, covering up to 15% of the total body surface area at a time. Additionally, burns treated with enzymatic debridement were found to be more superficial than initially assessed, highlighting its safety in preserving adjacent normal tissues. This procedure can be done directly at the patient’s bedside under regional nerve blocks or intravenous sedation, requiring only one operator, making it a practical and efficient option in burn care [27,28,29].

## 2. Clinical Indications and Use in Burn Management

Choosing between surgical and enzymatic debridement using NexoBrid^®^ for burn wounds involves several critical factors that influence the effectiveness and appropriateness of each method, as mentioned in Table 1.

Regarding the burned surface area, surgical debridement is often preferred for larger areas due to its ability to quickly remove extensive necrotic tissue, while enzymatic debridement may be more suitable for smaller or more localized burns [42]. Current indications of enzymatic debridement using NexoBrid^®^, as stated by the regulatory guidelines from EMA and FDA, are limited to burn wounds of up to 15% of the total body surface since data on large-area applications are limited. However, studies suggest NexoBrid^®^ can be reapplied afterward if more extensive debridement is needed [43]. Additionally, recent studies explore the use of enzymatic debridement in burns over 15% TBSA. A study reported by Hofmaenner et al. showed that enzymatic debridement using NexoBrid^®^ is safe for burns exceeding 15% TBSA, with no significant hemodynamic or inflammatory effects observed [44].

The anatomical location of the burns can affect debridement decisions. In patients with burns exceeding 15% TBSA, circumferential limb burns are prioritized for treatment, with other areas addressed based on ease of application and patient positioning. For those undergoing surgical excision of full-thickness burns, enzymatic debridement is performed concurrently, with NexoBrid^®^ applied and left in place during the surgical tangential excision of the full-thickness burns [45].

**Figure 1:**
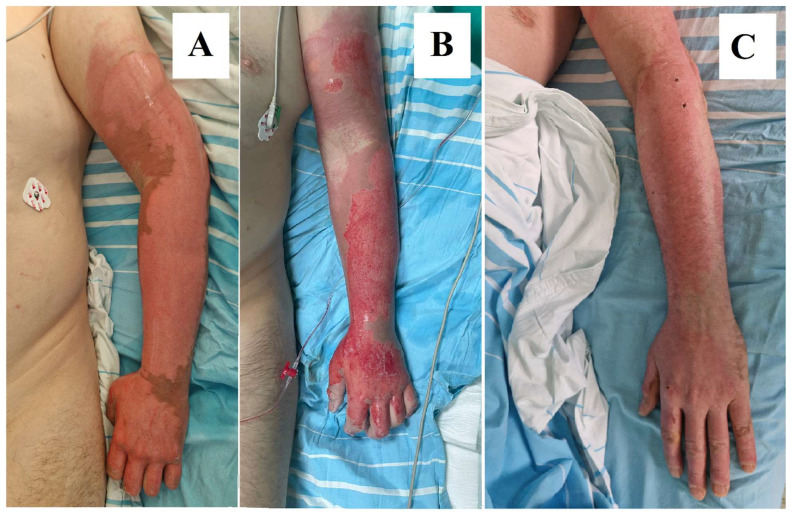
(**A**) Burn on arrival. (**B**) Wound bed after 4h of debridement using Bromelain. (**C**) Complete epithelization at 21 days after burn injury. Images are from our Burn Center and patient consent was obtained for the use of his case for scientific purposes.

The depth of the burn, ranging from superficial to full-thickness burns, determines the method of debridement. In deep partial-thickness burns, selective debridement with NexoBrid^®^ preserves the uninjured dermis, which is the major advantage of its use. In contrast, full-thickness burns may require more aggressive surgical debridement to remove all necrotic tissue [28,31,46] (Figure 1).

**Figure 2:**
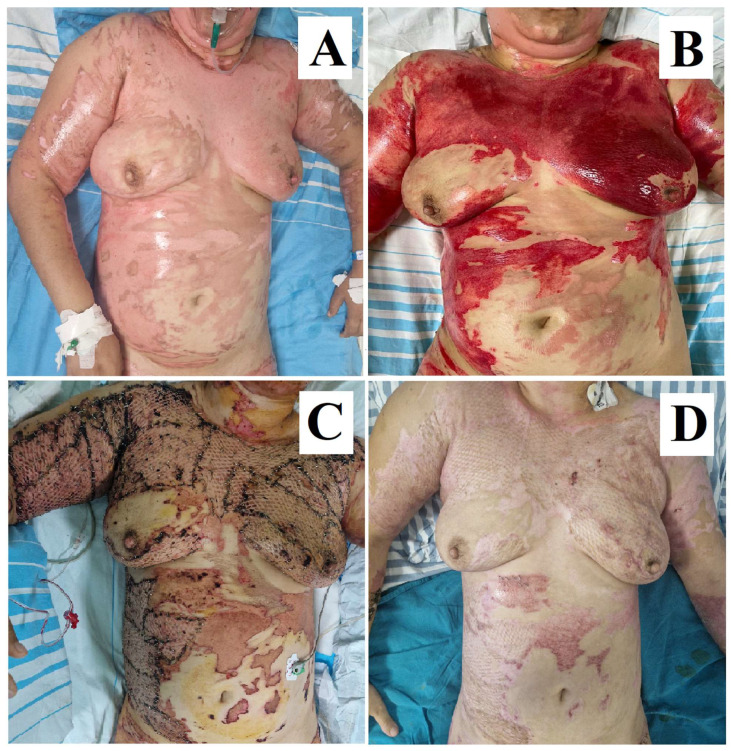
(**A**) Burn on arrival. (**B**) Wound bed after 4 h of debridement using Bromelain. (**C**) Day 7 after skin grafting. (**D**) Esthetic and functional outcome at 3 weeks after burn injury. Images are from our Burn Center and patient consent was obtained for the use of her case for scientific purposes.

**Figure 3:**
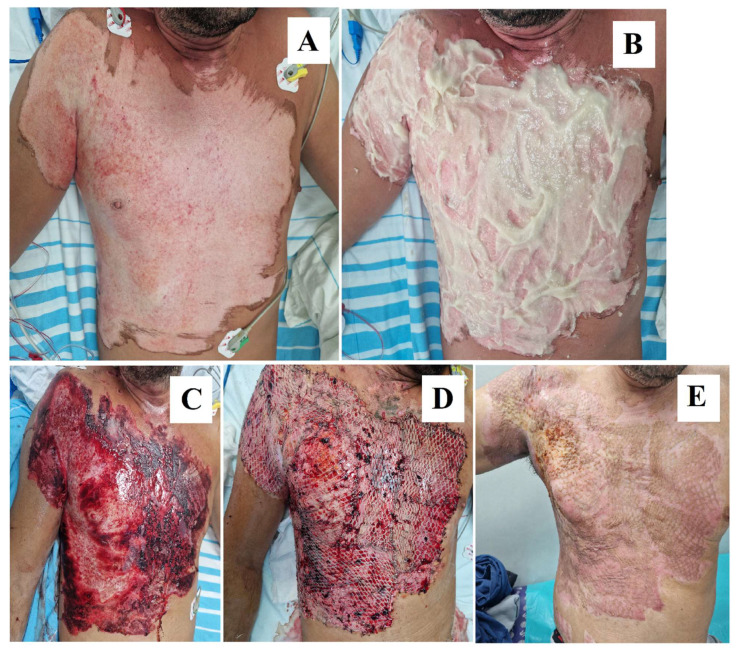
(**A**) Burn wound aspect on the second day after injury. (**B**) NexoBrid^®^ application at the bedside the second day after burn injury. (**C**) Wound bed after 4 h of debridement. (**D**) Day 1 after skin grafting. (**E**) Esthetic and functional outcome 1 month after skin grafting. Images are from our Burn Center and patient consent was obtained for the use of his case for scientific purposes.

Through a histological assessment performed pre- and post-debridement on partial-thickness burn wounds, Di Lonardo et al. showed that NexoBrid^®^ offers a key advantage by preserving the partially damaged dermis during the debridement process, which is critical for wound healing. This partially viable dermis, described as “homogenized”, retains enough structure to act like a natural scaffold, supporting the gradual reepithelization and recovery of the wound. If this layer is well-protected and managed appropriately—by avoiding desiccation and preventing infection—it can progressively regenerate, leading to spontaneous wound healing with improved morphological outcomes. However, if this dermal layer is not properly managed or if the underlying dermis is too damaged, particularly in deeper wounds or areas with thinner skin, surgical intervention such as skin grafting is needed to restore proper healing, as seen in Figure 2 and Figure 3 [47].

Regarding the presence of necrotic debris, the amount and consistency of necrotic tissue can influence the choice between surgical and enzymatic debridement. In their study, Bowers et al. recommend excluding patients with leathery, stiff eschars from enzymatic debridement using NexoBrid^®^, while recommending surgical excision. On the opposite, mid- or deep dermal burns, even those with large, pale white areas that are soft to the touch, are considered ideal candidates for enzymatic debridement using NexoBrid^®^ since with NexoBrid^®^ there is a greater potential for dermal preservation than with tangential excision [45].

A study by Sharaf et al. showed that the bacterial colonization of burns treated with NexoBrid^®^ is similar to other burns at all stages of wound healing, with the predominance of Gram-positive bacteria in the first week, followed by a predominance of Gram-negative bacteria in the second week [48]. Having the burn wounds debrided with NexoBrid^®^ leads to a slight decrease of infection rates through the removal of necrotic tissues and not due to an antimicrobial effect [49]. Using NexoBrid^®^ on old and infected burn wounds can lead to a systemic febrile response, transient bacteremia, or sepsis [40].

According to the latest European consensus guideline, the application of NexoBrid^®^ to the burn wound can be done immediately (in the first 12 h after burn injury), early (in the first 12–72 h), and delayed (after 72 h after burn injury) [24]. Enzymatic debridement is usually employed within the first 24–48 h after the burn injury [50].

The type of ointments or dressings previously applied to the burn can affect the choice of debridement, since some agents can reduce the effectiveness of enzymatic debridement, leading to the need for surgical treatment in these cases. Previous studies investigated in vitro the efficiency of debridement using NexoBrid^®^, when NexoBrid^®^ was applied after various common local agents used in burn treatment. It is advisable to use polyhexanide-containing agents such as Prontosan^®^ for rinsing and presoaking the wounds, as they do not significantly impair the enzyme’s activity at lower concentrations. The use of agents that contain silver or copper is not advisable when enzymatic debridement is planned, as these substances have been shown to decrease NexoBrid^®^’s enzymatic function, particularly at higher concentrations of silver or copper. Additionally, while NexoBrid^®^ remains effective across a range of pH levels, extreme pH values of 3 and 11 have been found to partially reduce its activity. Therefore, avoiding these extremes is recommended to ensure the best possible debridement outcomes [51].

However, the application of NexoBrid^®^ is time-consuming because it requires both preparation of the burn wound before application, 4 h associated with debridement, and the time needed for removal after application. In a study by Claes et al. on 67 patients, the mean time of the procedure was 486 min, with a range between 307 and 1796 min [52].

The use of surgical or enzymatic debridement using NexoBrid^®^ depends on the availability of resources and the expertise of the burn specialists leading the treatment. Surgical debridement requires specialized surgical teams, surgical instruments, and operating rooms, while enzymatic debridement requires access to NexoBrid^®^ and trained personnel to apply and guide the treatment effectively [53]. Individual patient considerations, such as overall health, comorbidities, and response to pain, also impact the choice. Enzymatic debridement may be preferred for patients who are at higher risk of complications from invasive procedures or who require less aggressive treatment methods [11,54].

A preliminary cost analysis from a burn center in Italy comparing surgical excision and covering with autografts to enzymatic debridement showed a reduction in costs of 5330 euros per patient in the group of patients debrided with NexoBrid^®^, suggesting a notable economic benefit of using NexoBrid^®^ for managing intermediate or intermediate–deep burns [41]. Another study by Kern et al. suggested that using NexoBrid^®^ to debride burn wounds at the bedside avoids the utilization of operating rooms, which further leads to increased reimbursements from other surgical cases [55].

## 3. NexoBrid^®^ Application and Procedure

Before applying NexoBrid^®^, it is essential to properly prepare the burn wound by cleansing it to remove any keratin or blisters, followed by hydrating the area with chlorhexidine-soaked gauze or liquid Prontosan^®^. A sterile paraffin ointment or Vaseline should be applied around the wound to protect the surrounding healthy skin from inadvertent exposure to NexoBrid^®^, though the product itself does not harm intact skin [31,56].

Bromelain powder is mixed with an inert carrier gel for easy application, with products on the market containing either 2 g of powder in 20 g of gel for debridement of 1% of the total body surface area or 5 g of powder in 50 g of gel for debridement of 2.5% TBSA [27]. Afterward, the NexoBrid^®^ gel is applied in a 1.5–3 mm-thick layer over the burn area, ensuring that it is fully contained within an occlusive dressing to prevent air pockets from forming and the gel from spreading outside the targeted wound area. After four hours, the dressing and adhesive barrier are removed and the dissolved eschar is gently scraped away. The wound is then cleaned with sterile gauze and saline until all tissue debris is removed. A wet-to-dry dressing is recommended post-debridement, so an antibacterial solution-soaked dressing should be applied over the wound for another 2 h, such as chlorhexidine-soaked gauze, polyhexanide gel, and paraffin gauze. The debrided area must be covered to prevent desiccation, the formation of pseudoeschar, and infection by applying a permanent or temporary skin substitute, such as Suprathel^®^, Biobrane^®^, or Mepitel^®^, or allografts in cases of burn wounds showing vital dermis and good healing potential or autografts in deep burns requiring surgical treatment [31,52,56,57,58].

The mechanism of action of NexoBrid^®^ is based on the presence of a concentrate of proteolytic enzymes that target necrotic burn eschars. After the application of NexoBrid^®^, burns that were initially considered to necessitate skin grafting based on their clinical aspect may prove themselves more superficial, with enough dermis to support spontaneous healing [27].

Even for experienced burn surgeons, a learning curve exists in interpreting the wound bed after enzymatic debridement using NexoBrid^®^ to determine whether grafting is needed or if spontaneous healing and epithelial regeneration will occur. The decision to proceed with grafting or allow for spontaneous healing often depends on several factors, including the appearance of the wound bed, the presence of viable tissue, and the dynamics of bleeding points [59,60].

According to Palao et al. [58], the depth of the burn can be assessed after enzymatic debridement using NexoBrid^®^ and classified into four types which guide the subsequent treatment. In type I, the visualization of a dermic wound bed with a multitude of small diameter pin-point bleeders (uniform shades of red to pink) corresponds to a superficial partial-thickness burn. In type IIa, the visualization of a scattered pattern of larger-diameter bleeders (irregular shades of pink to white) but without the loss of tissue thickness observed macroscopically corresponds to an intermediate/deep partial-thickness burn. In type IIb, the visualization of a scattered pattern of larger diameter bleeders (no shades and white color), with the loss of tissue thickness observed macroscopically, corresponds to a deep dermal burn. In type III, the visualization of fatty tissue corresponds to a full-thickness burn. Type I and IIa can heal by spontaneous epithelization in 30 days, while type IIb and III should be covered with skin autografts after 3–4 days following Bromelain-based debridement [58].

However, determining the precise timing and the need for surgical intervention following using NexoBrid^®^ can be highly challenging. Claes et al. recommend evaluating the wound bed twice: first, immediately after removing the NexoBrid^®^ to assess its viability, and again after a 2 h wet-to-dry dressing period to make a more informed decision. The authors identified several indicators for the need for skin grafting, including incomplete debridement after the application of NexoBrid^®^, a dermal step-off in the wound bed, the presence of visible fat lobules and translucent fat lobules, and/or the presence of visible blood vessels or coagulated blood vessels. In addition, combining laser Doppler examination with the identification of these wound bed characteristics can be used to make a decision [61].

Another study by Claes KEY et al. showed a marked reduction in the number of surgical procedures and the extent of autografting required for burn wounds as assessed by laser Doppler imaging, concluding that NexoBrid^®^ can effectively minimize the need for skin grafts. In this study, 39% of the deep burn areas as confirmed by laser Doppler imaging, which would have traditionally required surgery, healed conservatively without the need for grafting [62].

Mataro et al. compared the clinical assessment of burn depth before and after enzymatic debridement using NexoBrid^®^ and demonstrated that in the initial assessment of burns by an expert surgeon, the need for grafting was considered much more frequently compared to the evaluation of burns by the same surgeon after the application of NexoBrid^®^. Enzymatic debridement allowed for a more accurate diagnosis of burn depth, avoiding unnecessary surgical interventions [63].

## 4. Current Experience from the Literature

As NexoBrid^®^ was approved only recently, the literature on its use is scarce, with a limited number of studies and reports available. The European consensus guidelines regarding eschar removal by bromelain-based enzymatic debridement are only in their second edition and require additional clinical data to clarify the indications and limitations of using NexoBrid^®^ [24]. Initial concerns in studies addressed the safety, pain management, availability, and costs associated with the application. Nonetheless, a series of studies in the literature are promising regarding the use of enzymatic debridement and the exploration of NexoBrid^®^’s efficacy in treating burn patients, particularly for burns in functional areas, such as the face, hands, and genitalia.

In the current stage of knowledge, NexoBrid^®^ is not recommended for chemical burns, scalds, electrical burns, burns in children, or for burns covering more than 15% of the total body surface area when addressed in a single session of enzymatic debridement. NexoBrid^®^ is approved for pediatric use as it offers a novel, non-surgical solution for managing severe burn injuries in this vulnerable patient population [64].

In addition, NexoBrid^®^ has proven ineffective in patients with burns on diabetic feet, as these individuals may experience the development of additional eschar and worsening of their wounds. In patients with diabetic food burns, the zone of stasis can progress to necrosis despite treatment, with a delayed clinical presentation; therefore, rapid debridement does not offer significant advantages [65].

Hand burns affect over 80% of burn patients, either as isolated burns or as part of a more extensive injury, and although they are not life-threatening, they can cause significant functional impairment and lower the quality of life [66]. Due to the complex anatomy, surgical excision is technically challenging and can result in the loss of viable tissue, which, depending on burn depth, can lead to suboptimal aesthetic and functional outcomes. Bromelain-based debridement is more effective than tangential debridement, particularly in areas with thin skin and complex contours, such as the hands. In these regions, preserving as much viable dermis as possible during debridement leads to spontaneous epithelialization and improves aesthetic outcomes [67]. A study by Dadras et al. showed that in the case of deep partial-thickness burns, selective debridement with NexoBrid^®^, followed by local coverage with Suprathel, facilitates the spontaneous epithelialization of hand burns within the first 28 days after burn injury [60]. A study by Wu et al. on burned hands adds to the growing evidence that Bromelain-based debridement decreases the number of surgical interventions required for deep burns of the hand by allowing the surgeon to assess the burn wound more clearly and favors more conservative treatment strategies. Additionally, in their study, with the use of enzymatic debridement, escharotomies of the burned hands were avoided [33].

Facial burns cause significant aesthetic and functional damage. Studies have shown that the use of enzymatic debridement with bromelain improves scars by preserving viable tissue, leading to good outcomes for patients healing by spontaneous epithelialization and also for patients who require the use of autografts [43,68]. By maximizing the preservation of viable tissue, enzymatic debridement using NexoBrid^®^ also reduces the need for skin grafting. A study by Sampietro-De-Luis et al. on patients with partial-thickness and full-thickness facial burns showed complete epithelialization without the need for autografting in an average of 13.8 days and none of the patients included in the study presented ocular or infectious complications [69]. A comparison of scar quality between patients who underwent enzymatic debridement using NexoBrid^®^ and surgical debridement conducted more than 12 months after the burn showed the advantages of bromelain-based debridement, with improvements in pigmentation, thickness, relief, pliability, surface area, stiffness, and irregularity of the facial scars. However, there were no significant differences observed in erythema, melanin levels, viscoelasticity, transepidermal water loss, hemoglobin levels, or microcirculation between the two groups of patients [29].

A different possible application that emerged was the treatment of burn wounds in the genitalia and perineum, taking into account the importance of preserving tissue in this complex region and the risk of further loss associated with tangential excision. A study by Schulz et al. underscored the high selectivity in enzymatic debridement using NexoBrid^®^ that saves vital tissues, leaving enough dermis in place so that reepithelization can occur spontaneously and the need for autografting can be reduced. The risk of infection of burn wounds in the genitalia and perineum might be reduced with the use of NexoBrid^®^ [70].

Another potential indication for NexoBrid^®^ is the prevention of burn-induced compartment syndrome. There is growing evidence that NexoBrid^®^ can be useful for preventing burn-related compartment syndrome in circumferential burns of the extremity or extensive burns of the trunk. A study by Mataro et al. analyzed the intra-compartmental pressure recorded before and after the use of NexoBrid^®^, showing that the excessive pressure was reduced by 60% after one hour of application and the pressure returned to below 30mmHg, achieving complete debridement in 4 h [71]. On the contrary, a study by Grunherz et al. showed that a surgical escharotomy was preferred in severely burned patients with large full-thickness burns or in scalds. Surgical escharotomy is safer and more reliable in preventing burn-related compartment syndrome, especially in patients needing large volumes of fluid during the resuscitation phase, which is associated with important edema. Another advantage of surgical escharotomy is the fast release of pressure in the burned extremity, while enzymatic debridement is limited to burn surfaces <15% TBSA, requires time for application and an additional 4 h, and has the risk of insufficient eschar removal [72].

While initial trials limited NexoBrid^®^ use to 15% TBSA due to concerns about the effect of bromelain on coagulation, emerging evidence and expert consensus suggest that enzymatic debridement using NexoBrid^®^ can be safely extended to larger burn areas, potentially up to 30% TBSA, on an individual basis. However, further research is needed to substantiate its safety and efficacy in these larger debridement areas [73,74].

Other studies have raised the concern that the application of NexoBrid^®^ can increase the systemic inflammatory response in critical patients and, in some cases, cause transient hemodynamic instability. A recent Spanish multidisciplinary consensus recommends that critically ill patients should be hemodynamically stabilized, and hypovolemia should be corrected before the enzymatic debridement using NexoBrid^®^, except for circumferential burns when NexoBrid^®^ is used to prevent the development of compartment syndrome and avoid the need for escharotomy. In critical patients, the recommendation includes the application of NexoBrid^®^ starting with surfaces covering less than 15% of the total body surface area, and repeated as soon as the patient’s hemodynamic condition permits, with further applications as needed [75].

Studies on animals have raised a concern regarding the potential for coagulation abnormalities and increased risk of bleeding associated with enzymatic debridement. Higher doses of bromelain in rats have been linked to prolonged prothrombin and activated partial thromboplastin, increasing serum fibrinolytic activity and inhibition of the fibrinogen synthesis, as well as a direct degradation of fibrin and fibrinogen [74,76]. In addition, Martin N et al. reported a case of a patient with severe burns who developed coagulation abnormalities after the application of NexoBrid^®^. Based on the observations in their patient, the authors recommended monitoring for potential coagulation-related adverse effects in patients treated with NexoBrid^®^ and suggested using this product with caution in patients who are receiving antiplatelet or anticoagulant therapy, particularly if locoregional anesthesia techniques are employed during the enzymatic debridement procedure [77]. Thrombocytopenia in burn patients can result from several factors, including hemodilution caused by substantial fluid resuscitation in the early stages of the burn injury, platelet activation and consumption due to severe tissue damage and inflammation, suppression of bone marrow function, the effects of medications like antibiotics, disseminated intravascular coagulation, and sepsis [78,79,80]. Concurrently, the hypercoagulable state induced by severe burns—characterized by elevated levels of procoagulant factors and a systemic inflammatory response—heightens the risk of venous thromboembolism in burn patients [81,82]. A study by Capitelli-McMahon et al. showed no coagulation abnormalities associated with enzymatic debridement when compared to surgical excision [83]. The impact of enzymatic debridement with NexoBrid^®^ on coagulation requires further investigation to ensure patient safety.

Pain seems to be a significant barrier to the broader adoption of this technique. The European consensus on NexoBrid^®^ states that effective pain management should be regarded as a standard of care [24]. A study by Buta et al. examined pain management strategies for patients undergoing the application of NexoBrid^®^ and assessed pain levels using the Numeric Pain Rating Scale in adult patients with acute deep partial-thickness and full-thickness burns covering less than 30% of their total body surface area. The enzymatic debridement using NexoBrid^®^ was performed within 24 h of injury, with pain management conducted in line with European consensus guidelines. For burns on the upper or lower extremities, regional nerve blocks, safely administered using ultrasound guidance, were used to avoid the need for intubation and sedation. For smaller burns, such as isolated hand injuries, local blocks were applied. In cases of larger burns, analgosedation with ketamine or IV opiates combined with benzodiazepines (classified as conscious sedation, or CS) was utilized. The study aimed to optimize procedural pain control, addressing the slow adoption of NexoBrid^®^ in some centers due to concerns over pain management [38]. Other studies also reported intravenous sedation was sufficient in most patients where enzymatic debridement was used [84,85]. With the application of NexoBrid^®^, pain levels are highest shortly after the application and again during the removal, and should be addressed accordingly [52,85]. In a study by Schiefer et al. regarding patients’ satisfaction with anesthesia, analgosedation and regional blocks were found superior to other anesthesia techniques [39]. In patients with upper or lower limb burns, either unilateral or bilateral, regional anesthesia at the bedside is adequate for NexoBrid^®^ debridement and it’s a safe procedure, with fewer risks than general anesthesia [52].

NexoBrid^®^, initially developed for burn care, has paved the way for new applications in wound management, including chronic wounds. The successful outcomes with bromelain in burn patients led to an exploration of bromelain’s potential in treating chronic wounds. In a preliminary study, patients with at least one necrotic chronic wound—defined as non-healing and covered by eschar—(such as venous insufficiency ulcers, diabetic foot ulcers, pressure ulcers in the heel, or arterial insufficiency ulcers following revascularization) were treated with a 10% concentrate of proteolytic enzymes enriched in bromelain for several 4 h sessions. These preliminary results demonstrated that bromelain-based enzymatic debridement had potential efficacy and safety in chronic wounds, proving more effective for debriding moist eschars but less so for dry eschars [86]. Despite these promising results, the 4 h application time used in these studies posed limitations for home use and elderly patients. In response, this prompted the development of EscharEx^®^ (MediWound Ltd., Yavne, Israel), which utilizes bromelain to cleave and remove necrotic tissue, thereby preparing the wound bed for healing. EscharEx^®^ is intended for topical application over the wound bed, requiring several 24 h applications until the necrotic tissue is removed and the wound is fully debrided. The product is formulated for use with 4–8 daily applications to debride chronic wounds, promote granulation tissue, and reduce biofilm and bacteria [87,88,89]. This progression from burn treatment to chronic wound management highlights the adaptability of bromelain-based debridement in addressing a wider range of clinical needs.

## 5. Conclusions

The management of severe burn injuries continues to be challenging, despite significant advances in research and clinical practice. Enzymatic debridement using NexoBrid^®^ has shown considerable potential as a complementary approach to conventional surgical techniques, offering a less invasive and more precise method for removing necrotic tissue. Through preliminary research, clinical studies, and international collaboration, this method represents a promising step forward in enhancing burn care outcomes. However, it requires further studies and the expansion of its indications to confirm its efficacy and fully explore its potential in improving burn care. Ongoing evaluation and integration into clinical protocols will be essential to fully realize its benefits in specialized burn treatment.

## Figures and Tables

**Table 1:** Comparison between surgical and enzymatic debridement using NexoBrid^®^ [24,25,30,31,32,33,34,35,36,37,38,39,40,41].

Indication	Surgical Debridement	Enzymatic Debridement (NexoBrid^®^)
Burn depth	Used for deep burns or when rapid removal of necrotic tissue is needed. Recommended for full-thickness (third-degree) burns and deep partial-thickness burns.	Indicated for removal of burn eschar in adults with deep partial- and full-thickness thermal burns. Best for intermediate and deep dermal burns.
TBSA	Suitable for any burn surface. In extensive burns, multiple excision sessions may be required.	Current indication for 15% TBSA in one session. It was used off-label for higher burned surfaces.
Moment of debridement	Typically done as early excision in the first 72 h after burn injury.	Can be applied within 72 h after injury, requiring that the burn is properly assessed.
Speed of eschar debridement	Rapid through immediate surgical intervention: tangential excision, fascial excision, VERSAJET hydrosurgery system when available.	Slower, debridement is produced within 4 h of application, after burn wound preparation.
Control of debridement	Removal of necrotic tissue is performed by the surgeon in one session or as multiple subsequent sessions, required in extensive burns.	NexoBrid^®^ allows for selective removal of non-viable tissue, preserving viable tissue, in one session or as multiple subsequent sessions.
Tissue selectivity	Less selective: viable tissue is always sacrificed. Excision might be difficult in complex anatomic regions.	Highly selective; targets only necrotic tissue while preserving healthy tissue.
Indications in infected burns	Ensures rapid removal of infected tissue.	Concerns regarding pyrexia, transient bacteremia and sepsis.
Scarring and functional outcome	May lead to more significant scarring due to the excision of both necrotic and some viable tissue.	Less scarring due to selective tissue removal and preservation of dermal layers.
Anesthesia requirement	Depends on localization and extension of burns.	Depends on localization and extension of burns.
Resource needed	Requires a surgical theater, specialized equipment, and post-operative care.	Can be performed in a less resource setting than operative theater, typically requiring fewer resources than surgery. Can be performed at the bedside.
Cost	Surgical procedure costs.	Additional costs because requires the availability of NexoBrid^®.^

## Data Availability

This paper is a literature review; no new data were created.
